# Lack of health maintenance examinations and risk in myeloma patients

**DOI:** 10.1002/cam4.716

**Published:** 2016-04-27

**Authors:** Joseph D. Tariman, Charise Gleason, Beth Faiman, Deborah Doss, Donna Catamero, Jessica Bishop‐Royse, Mike Katz, Sandra Kurtin, Diane Moran, The IMF Nurse Leadership Board, Sagar Lonial

**Affiliations:** ^1^ College of Science and HealthDePaul UniversityChicagoILUSA; ^2^ Myeloma ProgramEmory UniversityAtlantaGAUSA; ^3^ Myeloma ProgramCleveland Clinic FoundationClevelandOHUSA; ^4^ Jerome Lipper Institute of MyelomaDana Farber Cancer InstituteBostonMAUSA; ^5^ Mount Sinai HospitalNew YorkNYUSA; ^6^ Social Science Research CenterDePaul UniversityChicagoILUSA; ^7^ International Myeloma FoundationLos AngelesCAUSA; ^8^ International Myeloma Foundation Nurse Leadership BoardLos AngelesCAUSA; ^9^ Hematology‐Oncology DepartmentUniversity of ArizonaTucsonAZUSA

**Keywords:** Cancer, health maintenance, health promotion, multiple myeloma, preventive health services, survivors

## Abstract

Health maintenance (HM) practices are essential to prevent illness, promote well‐being, and maximize health. Patients with multiple myeloma (MM) are at increased risk for cardiovascular disease and cancers, yet, research on HM practices and preventative care of MM survivors has limited report. The study comprised a descriptive, correlational, and cross‐sectional online survey design. Survey of patients with MM was carried out through the International Myeloma Foundation (IMF) and the Association of Cancer Online Resources (ACOR) e‐mail list services. The members of the IMF and ACOR e‐mail list services were surveyed, of which 237 patients responded. The modified Medical Expenditure Preventive Survey–Preventive Care questionnaire was used; it included items that ask patients regarding their healthcare practices that relate to dental care, cancer prevention, addiction, lifestyles, sensory screening, immunizations, cardiovascular, endocrine, psychosocial, and bone health. Descriptive statistics, Pearson's chi‐square, and Spearman's rho correlation coefficient were obtained. In this study, men had statistically significant inferior global health maintenance scores than women (*P* = 0.002). Being employed (*P* = 0.054) and married or partnered (*P* = 0.017) were significantly correlated with better health maintenance patterns among male respondents. In contrast, no statistically significant correlations between sociodemographic factors and health maintenance patterns were found in women. Patients with MM, particularly men, require continued education and close monitoring of health maintenance practices. These findings are consistent with publications looking at gender disparities in healthcare utilization in the United States. Studies show that men, in general, are less likely to seek preventative healthcare screenings. Healthcare providers must incorporate health maintenance promotion during clinic visits.

## Introduction

Multiple myeloma (MM) is a malignant disorder of the bone marrow plasma cells, which can present with many clinical manifestations. While there may be small subsets of patients who achieve long‐term durable remissions, most patients will relapse and eventually die of their disease 1. Recent data suggest that there are approximately 70,000 persons living with MM in the United States with an average age at diagnosis of 70 years 2. Overall survival for patients with MM has improved significantly over the last decade with a median expected overall survival of between 7 and 10 years, which is primarily attributed to the use of novel agents to treat MM as well as improvement in the prevention and management of disease‐ and treatment‐related adverse events 3. However, novel agents used to treat MM are not without adverse events, which in certain instances may cause or exacerbate comorbidities, may limit treatment options, or affect quality of life 4.

The leading causes of death for patients with MM are infections, in particular pneumonia 5. Bone disease, renal damage, hematologic toxicities, infections, thromboembolism, and peripheral neuropathy are the most frequent disabling events requiring prompt and active supportive care 6. The most common comorbidities noted in the literature for patients with MM include cardiovascular disease (coronary artery disease, hyperlipidemia, and hypertension), chronic lung disease, and endocrine disorders including diabetes and arthritis 7. Thus, health maintenance (HM) and wellness promotion are critical to preserving treatment options and limiting morbidity and mortality in the patient with MM.

Given the improved survival and well‐being for the majority of patients with MM, together with the expectation of long‐term episodic care with inherent risks of adverse events, it is important to ensure that HM and preventive care (PC) practices are incorporated into the standard of care for all patients with MM. However, there are few descriptions or analyses that detail if this is in fact occurring, and if so, what is being found. Moreover, a review of the literature on HM shows little emphasis on HM and PC in the myeloma patient population 8.

Several studies have identified that both men and women are at a higher risk for heart disease if risk factors exist such as elevated blood pressure, the presence of cigarette smoking, elevated total cholesterol, elevated low‐density lipoprotein, and low levels of high‐density lipoprotein 9, 10, 11. Hyperlipidemia, inactivity, and adiposity can lead to early cancer‐related mortality 12, 13, 14. Sociodemographic variables such as age, gender, race, unemployment, and partner status have been studied as potential determinants to HM and PC services utilization among patients diagnosed with cancer. Yood et al. 15 reported that among women with similar medical care access before their diagnoses, there are ethnic differences in stage of breast cancer at diagnosis; African American women were diagnosed at a later stage than were European American women. Even after adjusting for age, marital status, income, and stage, the hazard ratio was still significant for race as a determinant for late diagnosis of breast cancer in African American women at 1.0 (95% CI = 0.7–1.5).

In 2006, Takeda et al. 16 reported that age and gender differences had an impact on multiple roles on health and health‐related behaviors; younger women benefited from multiple roles by smoking less, while younger men demonstrated more high‐risk behaviors by doing more smoking and heavier drinking. Interestingly, for middle‐aged men, they tend to smoke less and had fewer health problems, while middle‐aged women reported lower HM behaviors, exercising less and having a fewer health check‐ups. In persons with no cancer, race by gender heterogeneity has statistically significant difference in terms of contribution to development of multimorbidity among subpopulations based on the intersection of race and gender. Restless sleep in 2001 predicted chronic medical condition 10 years later in 2011 among Black women (standardized adjusted *B* = 0.135, *P* < 0.05) and White men (standardized adjusted *B* = 0.145, *P* < 0.01). Additionally, White women (standardized adjusted *B* = 0.171, *P* < 0.001), but not Black men (standardized adjusted *B* = 0.001, *P* > 0.05) had predicted chronic medical condition 10 years later 17.

No previous studies have been conducted to describe HM and PC practices in patients with MM. Insight into HM into this population is critical to modify risk factors for developing therapy‐associated cardiovascular and other preventable illnesses during the time one lives with MM. Thus, we have designed an anonymous Internet‐based survey with the following objectives:

1. To describe the HM and PC patterns in patients with multiple myeloma in the United States.
2. To determine if HM and PC patterns in patients with MM differ according to sociodemographic data including gender, age, income, education, relationship status, and employment status.

## Methods

### Design

The study utilized a descriptive, correlational, and cross‐sectional online survey design.

### Patients and setting

A convenience sample of patients from the International Myeloma Foundation (IMF) and ACOR's 1200 member e‐mail lists were recruited for this study. A total of 294 responded to the survey but only 237 patients (approximately 20% response rate) across the United States with a diagnosis of symptomatic multiple myeloma completed the survey (50% completion of survey or more). Table 1 outlines the sociodemographic information of study patients. The patient inclusion criteria include (1) adults 18 years of age and above; (2) diagnosed with multiple myeloma; (3) ability to read and write English; (4) ability to give informed consent. Patient exclusion criteria include adults diagnosed with lymphoma, leukemia, or monoclonal gammopathy of undermined significance. Patients diagnosed with a secondary malignancy of any type and currently receiving chemotherapy for a second malignancy were also excluded.

**Table 1:** Sociodemographics of survey respondents (*N* = 237)

Category	Response	%^a^	*N* a	Category	Response	%^a^	*N* a
Gender	MaleFemale	45.854.2	237	Age	30–3940–4950–5960+	2.16.827.064.1	237
Race	CaucasianAsianBlack	94.31.84.0	227	Relationship	SingleMarried/PartneredSeparatedDivorcedWidowed	3.483.50.88.04.2	237
Ethnicity	HispanicNon‐Hispanic	4.295.8	237	Income	<18K18–35K36–55K56–85K86K+Prefer not to answer	3.49.310.219.934.722.5	236
Work status	WorkingNot working	27.472.6	237				
Education (highest completed)	9–12th grade2 year collegeGraduate degree	13.116.929.140.9	237				
Have PCP	YesNo	92.87.2	235				

PCP, primary care physician.

a Valid responses.

### Survey questionnaire

The modified Medical Expenditure Preventive Survey (MEPS)‐PC questionnaire from the Agency for Healthcare Research and Quality (AHRQ) included items that ask patients regarding their healthcare practices that relate to dental care, cancer prevention, cardiovascular health, endocrine health, nutritional intake, addiction, lifestyles, bone health, sensory screening, psychosocial health, immunizations, and patient–provider encounter. MEPS‐PC questionnaire is a well‐validated instrument in measuring heathcare maintenance and preventive healthcare practices among adults dwelling in the community with myeloma specific modifications 18. Face validity of the questionnaire was tested among members and determined to be excellent, requiring very minimal revision in the questionnaire items. The questionnaire was derived from the critical appraisal of every item by each member of the IMF Nurse Leadership Board research team members.

### Recruitment procedure

After obtaining approval from Emory University Internal Review Board, the online survey was sent to IMF and ACOR e‐mail list members. Eligible patients completed a survey using the modified AHRQ's MEPS‐PC questionnaire and investigator‐developed sociodemographic questionnaire via the Internet. The total respondent burden in completing the survey did not exceed 30 min, with average completion time of 22 min. The online questionnaires were administered immediately after the cover letter was accessed by potential study patients. The patients were asked to complete the sociodemographic questionnaire at the end of the online survey.

### Analysis

The underlying hypothesis of this study states that there is no difference in the patterns of HM and preventive healthcare practices between dichotomous categories of sociodemographic variables (e.g., men vs. women; employed vs. unemployed; partnered vs. nonpartnered). In this study, a power analysis estimated that a sample of 282 subjects have 80% power to detect statistically significant differences between dichotomous groups at 0.05 alpha level using Pearson's chi‐square statistics. Spearman's rho was utilized to assess statistically significant correlations between adequate HM patterns and selected dichotomous sociodemographic variables. Each item on the MEPS‐PC were indexed into five main domains: recommended healthcare team member visits (primary care physician, dentists, ophthalmologist), adherence to vaccination (influenza, pneumococcal, and tetanus booster vaccination) guidelines, health education by primary care physicians (health education on diet, exercises, alcohol use), adherence to routine clinic tests (annual cholesterol and thyroid tests and stool examination for blood), routine screening procedures (DEXA bone density study, colonoscopy or sigmoidoscopy, and prostate examination for men; mammography, Pap smear, and self‐breast examination for women).

## Results

Two‐hundred ninety‐four potential subjects attempted to complete the online survey, but only 237 (22% overall response rates) completed more than 50% of the entire survey. Those with less than 50% completion were eliminated. The online survey was conducted from June to August 2014. In the sample, 46% were male and 52% female; 91% of respondents were age 50 and older; 86.9% of respondents were well educated and had completed college with a 2‐year, 4‐year, or master's degree; 95% were not Hispanic; and 94% identified themselves as being Caucasian, specifically. Table 1 presents additional sociodemographic characteristics of study patients.

### Recommended healthcare team visits

**Figure 1:**
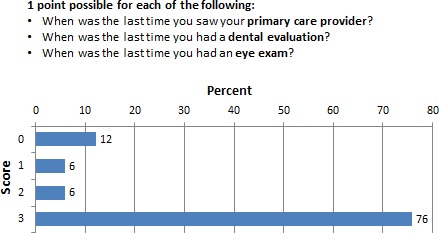
Percentage of respondents who scored 0–3 in the healthcare team subscale.

The survey asked patients to note the last time they saw their primary care physician (PCP), dentist, or ophthalmologist for routine healthcare visit. Responses that are adherent to AHRQ's recommendations and guidelines were coded as a score of 1, whereas those responses that were not in compliance with the guideline were coded as a score of 0. Figure 1 shows a majority of respondents (76%) were adherent to the recommended visits to PCP, dentist, or ophthalmologist.

### Adherence to recommended vaccinations

**Figure 2:**
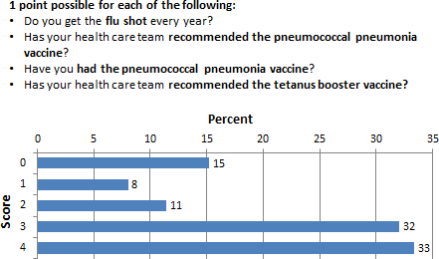
Percentage of respondents who scored 0–4 in the adherence to vaccination subscale.

When asked about their adherence to recommended vaccinations, 15% (*N* = 35) of the respondents did not have any of recommended vaccinations and only 33% (*N* = 78) were fully adherent to the recommended influenza, pneumococcal, and tetanus booster vaccinations. Moreover, approximately 8% (*N* = 19) of the respondents only had one of the three recommended vaccinations. Figure 2 shows the full details of the respondents' compliance to vaccination guidelines.

### Provision of routine health education by healthcare providers

**Figure 3:**
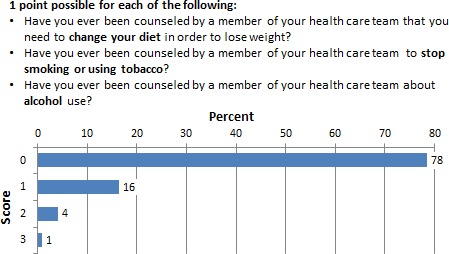
Percentage of respondents who scored 0–3 in the health education by healthcare practitioner subscale.

The respondents were asked whether or not they received routine health education related to diet, weight control, smoking, or alcohol abuse. Interestingly, 78% (*N* = 185) responded that they have not received health education on these topics. Only 1% of the respondents stated that they have received health education in topics related to diet, weight control, smoking, or alcohol abuse. Figure 3 demonstrates the full data on healthcare provider's provision of health education.

### Adherence to routine clinic tests

**Figure 4:**
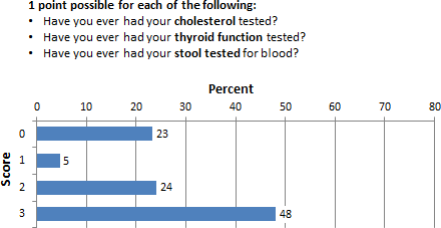
Percentage of respondents who scored 0–3 in the routine clinic test subscale.

Figure 4 shows that 23% (*N* = 57) of the respondents did not have any routine check for cholesterol, thyroid, or stool examination for blood test. Approximately 72% (*N* = 170) of the respondents had at least two of the three recommended routine clinic tests.

### Routine screening procedures: men and women

**Figure 5:**
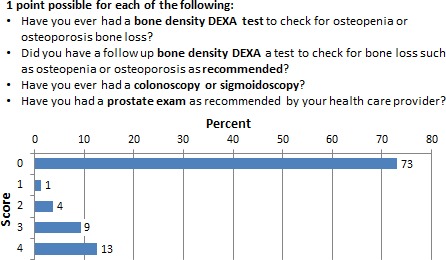
Percentage of respondents who scored 0–4 in the adherence to routine screening procedure subscale for men.

**Figure 6:**
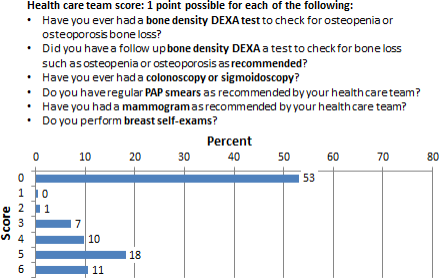
Percentage of respondents who scored 0–6 in the adherence to routine screening procedures for women subscale.

Routine health screening procedures were evaluated. Procedures for both men and women included bone densitometry and colonoscopy/sigmoidoscopy. Gender‐specific screening consisted of prostate examination (men) and pap smears, mammography, and breast self‐examination for women. Figure 5 shows 73% of male respondents never had the recommended routine screening procedures, whereas 53% women underwent the recommended testing for their age and sex as shown on Figure 6.

### Adequacy of HM: men and women

**Figure 7:**
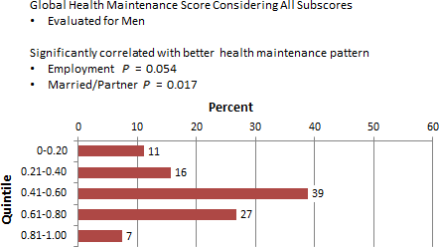
Percentage of male respondents by global maintenance score quintile.

**Figure 8:**
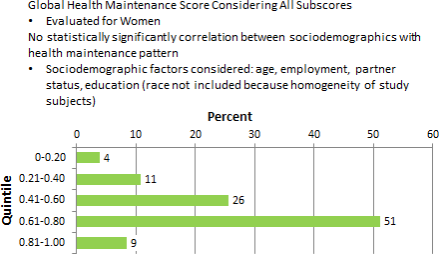
Percentage of female respondents by global maintenance score quintile.

**Figure 9:**
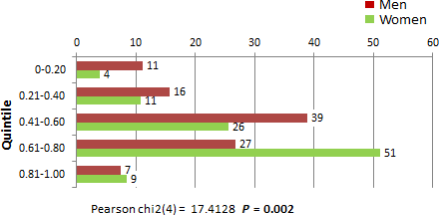
A comparison between male and female respondents in terms of global maintenance score quintile.

**Figure 10:**
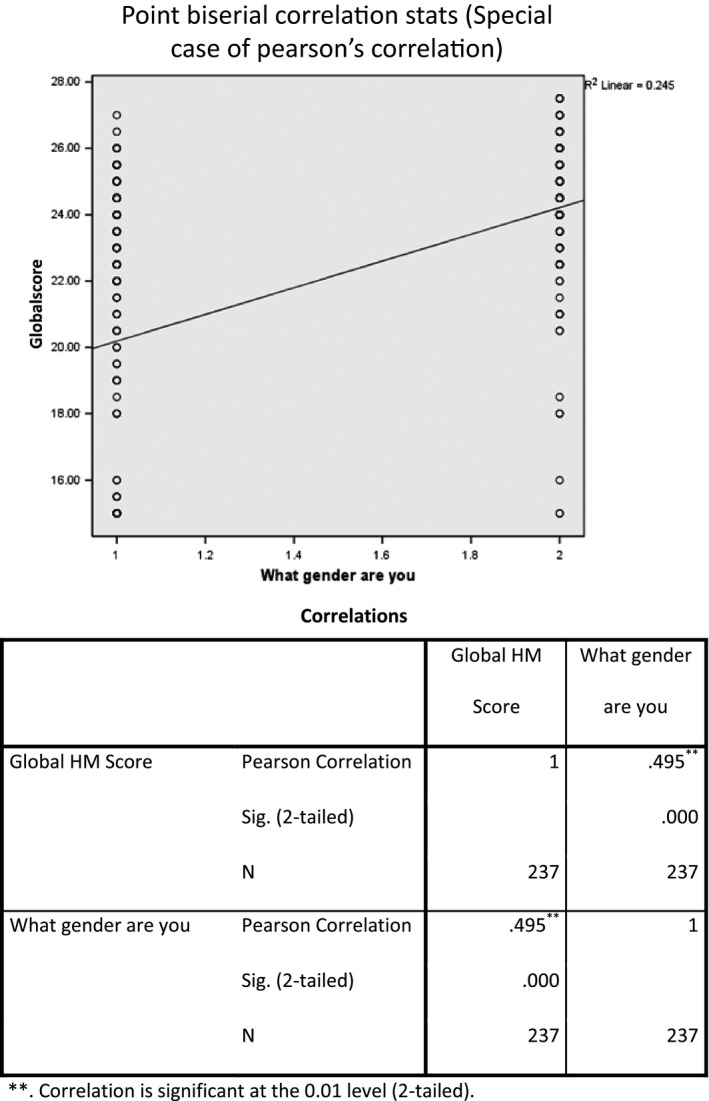
Point biserial correlation (a special case of Pearson's correlation coefficient) showed statistically significant positive correlation (*P = *0.000) between women and global health maintenance score.

The Global HM score, when considering all subscores, was evaluated for men and women. Overall, employment and partner status was identified as a factor in HCMP among men, but not for women. Figure 7 shows that being employed (*P* = 0.054) and married or partnered (*P* = 0.017) were significantly correlated with better HM patterns among male respondents. For women, sociodemographic factors such as age, employment, partner status, and education were considered. Race was not included because of the homogeneity of study subjects. There were no statistically significant correlations between sociodemographic factors and HM patterns (Fig. 8). Women in this survey had higher overall frequencies in HCMP than men (within the upper 60–80 and 81–100 quintiles). Figure 9 shows the global score for men versus women were statistically significant with men having poorer HM than women (*P* = 0.002). Figure 10 also showed statistically significant positive correlation between women and adequacy of HM.

## Discussion

Our results in terms of counseling for preventative services were consistent among other studies. Research has shown that cancer survivors receive less counseling by their primary care physicians on important health behaviors which have long‐term implications for health and include diet, exercise, and smoking cessation 18. This evidence underscores the need for oncology clinicians to also incorporate counseling related to healthcare maintenance and preventive health practices to their patients at every clinic encounter.

In this study, it is important to note that the study participants across sociodemographic factors did well with visits to their primary care physicians, dentists, and ophthalmologist. This is so important to monitor in myeloma patients because myeloma patients are at a higher risk of developing osteonecrosis of the jaw from bisphosphonates, cataracts, and glaucoma from prolonged use of steroids to treat their myeloma. Adherence to these health maintenance visits must be monitored regularly.

Low levels of health literacy have been correlated with lower socioeconomic and minority status, older age, and poorer health‐related outcomes especially in older individuals 19, 20, 21, 22. Thus, the investigators would anticipate that in this study, the respondents would have better HM patterns because the respondents have higher level of education and only a few were minorities. Research has shown that 74.8% of households in the United States have access to the Internet. Older individuals, such as patients with MM, are a rapidly growing demographic of Internet users searching for information, social networking, or e‐mail 23. The sample in this survey reflect these trends with 70% having a college degree or higher, and with obvious access to the Internet by virtue of their participation.

Research studies have investigated healthcare practices among cancer survivors. Breast cancer survivors when compared to matched controls were more likely to receive mammograms 24. Prostate cancer survivors were more likely to receive the influenza vaccination than controls; however, they were less likely to have had colorectal screening. Both the control group and prostate cancer group had similar rates of cholesterol and colorectal screening by 5 years post diagnosis 23. These findings are suggestive that a cancer diagnosis did not influence increased long‐term surveillance, but longitudinal studies are still needed to understand trends and patterns among myeloma survivors.

Colorectal cancer survivors were more likely to have screening services (mammogram, cervical, cholesterol, influenza, and bone densitometry) during the first year post diagnosis. Race and socioeconomic status were correlated with better HM; White patients were more likely to have received the flu vaccination than non‐White patients, and patients with a high socioeconomic status were more likely to have received the influenza vaccination, mammography, and cervical cancer screening 24. In this study, socioeconomic status has no correlation to any HM and PC services utilization.

A study in 2009 did find higher self‐reported screening rates among breast, prostate, and colorectal cancer survivors when compared to controls 25. Marital status was a significant predictor of preventive services. Married patients were more likely than controls to have had colorectal and prostate screenings. Similar results were demonstrated among MM patients in this study 26, 27, 28.

Health screening tests and health behaviors of patients with hematologic malignancies and had received hematopoietic stem cell transplantation had similar practices as to that of the controls that are age‐matched individuals without cancer 29. These findings as well as the findings in this study suggest that there is a lack of understanding about survivorship and the need for better healthcare maintenance pattern and preventive healthcare utilization in patients with cancer.

Health promotion and prevention are key elements of survivorship care. Health promotion includes risk avoidance (modifiable risk factors) and integration of elements of a healthy lifestyle including diet and exercise 30. Risk factors for MM include advanced age, male gender, obesity, and African American descent 31, 32. This survey did not capture any patients of African American decent, suggesting a population bias. Occupational data, physical data, including weight and body mass index, were not captured, limiting the direct implications for individualized health prevention and HM interventions.

A more recent literature review on cancer survivors and their health revealed that at least 50% of cancer survivors suffer from late treatment‐related side effects, often including physical, psychosocial, cognitive, and sexual abnormalities, as well as concerns regarding recurrence and/or the development of new malignancies. Researchers also found that some of their medical issues are chronic in nature and some are severe and even life‐threatening. Although sociodemographic variables were not examined closely by the authors of this review, the authors overwhelming found that all survivors, in general, face issues involving lack of appropriate HM, counseling, increased unemployment rate, and workplace discrimination.

Finally, clinicians must remember that during the first year of diagnosis, most myeloma patients are engaged in intensive therapy schedules and will likely forego some of the HM and PC tests. However, strong consideration to address HM and PC services must be initiated sooner than later to avoid consequences from lack of preventive care. A systematic mailing reminder can be instituted as well as phone calls and automatic text messaging for better compliance and adherence.

### Study limitations

The study results must be interpreted in light of several limitations of the sample selection and study design. First, surveys are a research method by which information is typically gathered by asking a subset of people questions on a specific topic. Internet‐based surveys are therefore limited to patients with access to the Internet. Although more patients have access to the Internet in their homes than in the past 33, certain populations are less likely to have Internet access and to respond to online questionnaires such as this. Thus, the population studied is only representative of a subset of Internet capable patients who use the Internet and are motivated to participate in a surgery. For these reasons, the sample is not necessarily representative of the general population.

The self‐selection factor/sample bias may further limit the findings. Participants in this survey may be viewed as highly motivated and interested in the topic of the survey, preventative health, and HM, thus further limiting the generalizability of the findings. As previously mentioned, survey responders were highly educated with limited ethnic diversity, not dissimilar to most convenience survey, but limiting the generalizability of the findings. The lack of heterogeneity should be addressed through future research as these findings are less generalizable across racial and ethnic groups.

Another consideration in the design of the study relates to content validity. Content validity is concerned with the adequacy and accuracy of items in an instrument, and the ability to measure what it is you intend to measure 34. It was observed retrospectively that some questions led to different assumptions and measured more than one construct. Future item development will fine tune the questions and ensure only one construct is measured for every question.

The small sample size is also a limitation to the generalization of the findings to the whole myeloma patient population. However, meaningful and statistically significant findings were still derived from the available data. We recommend that future studies will be adequately powered to 85% to account for possible attrition or invalid, partial, incomplete surveys.

## Conclusion

Patients with MM, particularly men, require continued education and close monitoring of HM practices. These findings are consistent with publications looking at gender disparities in healthcare utilization in the United States. In general, men are less likely to seek preventive healthcare screenings and services according to the U.S. Preventive Services Task Force 35. Healthcare providers must incorporate HM promotion during routine clinic visits.

## Conflict of Interest

None declared.
